# Identification of Human Gut Microbiome Associated with Enterolignan Production

**DOI:** 10.3390/microorganisms10112169

**Published:** 2022-10-31

**Authors:** Kento Sawane, Koji Hosomi, Jonguk Park, Kouta Ookoshi, Hinako Nanri, Takashi Nakagata, Yi-An Chen, Attayeb Mohsen, Hitoshi Kawashima, Kenji Mizuguchi, Motohiko Miyachi, Jun Kunisawa

**Affiliations:** 1 Central Laboratory Innovation Center, Nippn Corporation, 5-1-3 Midorigaoka, Atsugi 243-0041, Kanagawa, Japan; 2 Laboratory of Vaccine Materials, Center for Vaccine and Adjuvant Research, National Institutes of Biomedical Innovation, Health and Nutrition (NIBIOHN), 7-6-8 Saito-Asagi, Ibaraki 567-0085, Osaka, Japan; 3 Graduate School of Pharmaceutical Sciences, Osaka University, 1-6 Yamadaoka, Suita 565-0871, Osaka, Japan; 4 Laboratory of Gut Environmental System, Collaborative Research Center for Health and Medicine, NIBIOHN, 7-6-8 Saito-Asagi, Ibaraki 567-0085, Osaka, Japan; 5 Artificial Intelligence Center for Health and Biomedical Research, NIBIOHN, 7-6-8 Saito-Asagi, Ibaraki 567-0085, Osaka, Japan; 6 Department of Physical Activity Research, NIBIOHN, 1-23-1 Toyama, Shinjuku, Tokyo 162-8636, Japan; 7 Laboratory of Gut Microbiome for Health, Collaborative Research Center for Health and Medicine, NIBIOHN, 7-6-8 Saito-Asagi, Ibaraki 567-0085, Osaka, Japan; 8 Institute for Protein Research, Osaka University, 3-2 Yamadaoka, Suita 565-0871, Osaka, Japan; 9 Graduate School of Medicine, Osaka University, 2-2 Yamadaoka, Suita 565-0871, Osaka, Japan; 10 Graduate School of Dentistry, Osaka University, 1-8 Yamadaoka, Suita 565-0871, Osaka, Japan; 11 Graduate School of Science, Osaka University, 1-1 Machikaneyamacho, Toyonaka 560-0043, Osaka, Japan; 12 International Vaccine Design Center, The Institute of Medical Science, The University of Tokyo, 4-6-1 Shirokanedai, Minato-ku, Tokyo 108-8639, Japan; 13 Department of Microbiology and Immunology, Kobe University Graduate School of Medicine, 7-5-1 Kusunoki-cho, Chuo-ku, Kobe 650-0017, Hyogo, Japan; 14 Faculty of Science and Engineering, Waseda University, 2-2 Wakamatsu, Shinjuku-ku, Tokyo 162-8480, Japan

**Keywords:** gut microbiome, lignan, enterolignan, metabolism, machine learning

## Abstract

Dietary plant lignans are converted inside the gut to enterolignans enterodiol (ED) and enterolactone (EL), which have several biological functions, and health benefits. In this study, we characterized the gut microbiome composition associated with enterolignan production using data from a cross-sectional study in the Japanese population. We identified enterolignan producers by measuring ED and EL levels in subject’s serum using liquid chromatography-tandem mass spectrometry. Enterolignan producers show more abundant proportion of Ruminococcaceae and Lachnospiraceae than non-enterolignan producers. In particular, subjects with EL in their serum had a highly diverse gut microbiome that was rich in Ruminococcaceae and Rikenellaceae. Moreover, we built a random forest classification model to classify subjects to either EL producers or not using three characteristic bacteria. In conclusion, our analysis revealed the composition of gut microbiome that is associated with lignan metabolism. We also confirmed that it can be used to classify the microbiome ability to metabolize lignan using machine learning approach.

## 1. Introduction

Gut microbes have great impact on human health through the production of a variety of biologically active metabolites from dietary nutrients [1,2]. For example, short-chain fatty acids (e.g., acetate, propionate, and butyrate), produced through the microbial degradation of dietary fiber, interact with fatty acid receptors (e.g., GPR41 and GPR43) and play important roles in the prevention of intestinal diseases as well as metabolic disorders, such as obesity and diabetes [3]. The estrogen receptor-stimulating activity of soy isoflavone is enhanced through microbial conversion to equol, which plays an important role in the control of osteoporosis and menopausal symptoms [4,5]. Dietary polyunsaturated fatty acids also undergo microbe-dependent conversion into fatty acid metabolites [6,7], and we recently identified a microbial omega-3 fatty acid metabolite involved in the regulation of high fat diet-induced diabetic symptoms and skin inflammation by interacting with a peroxisome proliferator-activated receptor [8]. Microbial metabolites are physiologically active and contribute to the maintenance of bodily health; therefore, understanding gut microbial metabolism is an important topic in the health sciences.

As a method for evaluating associations among biological parameters (e.g., dietary nutrients, gut microbiome, impact on host health), machine learning system has been often used. Machine learning is one of the methods of data analysis, in which a machine (computer) automatically learns from the data and finds the rules and patterns behind the data. In previous studies, machine learning system, especially random forest, were used to predict target variable (e.g., disease-related factors) based on various explanatory variables such as dietary habit, gut microbiome, gene, and lifestyle [9]. For example, a cohort study succeeded in prediction of postprandial responses (e.g., blood triglyceride, blood glucose) to diet using random forest regression analysis with data including dietary habit, gut microbiome and host genetics [10]. A random forest regression analysis of 16S ribosomal RNA data identified the gut microbiome maturity of malnourished children [11] and these results were used to provide complementary food to children [12]. Productivity of short-chain fatty acids from dietary fiber was elucidated as a key factor of health benefits of the Mediterranean diet through a random forest classification analysis [13]. As such, machine learning-based evaluation system, especially random forest, have potential to predict the health efficacy of dietary nutrients whose metabolism is dependent on gut microbiome.

Plant lignans (e.g., pinoresinol, lariciresinol, and secoisolaricilesinol) are polyphenols that are present in various dietary sources (e.g., flaxseed, sesame, and sunflower seeds) and widely consumed worldwide [14]. Plant lignans are converted to enterolignans (enterodiol [ED] and enterolactone [EL]) through their intermediates, such as secoisolariciresinol (SECO) and didemethyl-SECO [15,16,17]. Enterolignans exert several bioactivities by interacting with estrogen receptors, such as estrogen nuclear receptor (ER) α, ERβ, and the G protein-coupled estrogen receptor (GPER) [18,19]. Therefore, the conversion of plant lignans to enterolignans is a key component of the health benefits of plant lignans. Indeed, a epidemiological study has suggested an association between blood concentration of enterolignans and lower risk of mortality from type 2 diabetes in Danish population [20]. Another study found an association between higher serum enterolactone and lower colorectal cancer-specific mortality in female [21]. The amount of plant lignan intake and plasma EL concentration were inversely associated with metabolic risk factors, such as triglycerides [22]. In an intervention study of dietary flaxseed-derived plant lignan on hypercholesterolemic Chinese male subjects, the blood cholesterol-reducing effect correlated with plasma enterolignan concentration [23]. These studies indicate that individual enterolignan metabolic profile determines both the physiological and health benefits of dietary plant lignan.

The conversion of plant lignans to enterolignans is mediated by intestinal bacteria [15,16]; not everyone can produce enterolignans in the gut owing to differences in gut microbiome [17]. Previous studies have indicated that people who can produce EL have highly diverse gut microbiome [24,25,26]. Although studies focusing on enterolignan-producing bacteria have reported some specific intestinal bacteria species capable of catalyzing the conversion of plant lignan to enterolignans (e.g., *Eggerthella lenta* for conversion to SECO, *Blautia producta* for de-methylation of SECO, *Gordonibacter pamelaeae* for dehydroxylation of didemethyl-SECO, *Lactonifactor longoviformis* for dehydrogenation of ED) [15], information on enterolignan-producing bacteria is still limited.

The gut microbiome differs between ethnicities, leading to differences in gut microbiome-dependent nutrient metabolism [27]. As such, it is expected that the characteristics of enterolignan productivity (including the percentage of people who can metabolize lignan) may differ between ethnicities. Indeed, the gut microbiome of the Japanese population is considerably different from that of people in other countries and has different genetic profiles involved in the metabolism of nutrients, such as carbohydrates and amino acids [27]. As Japanese population generally consume plant lignans [28], studies on the Japanese population may be helpful in making novel findings on the metabolism of dietary lignans by intestinal bacteria.

In this study, we evaluated the characteristics of the gut microbiome in Japanese population who can produce enterolignans. We identified individual enterolignan productivity by measuring their serum ED and EL levels using liquid chromatography-tandem mass spectrometer (LC-MS/MS), at the same time, we determined the composition of their gut microbiome using QIIME1. We detected the bacteria that are peculiar to ED and EL producers, and we use their abundance only to build machine learning model to classify the study subjects to EL producers or not.

## 2. Materials and Methods

### 2.1. Preparation of Enterodiol-Glucuronide (EDGlu) and Enterolactone-Glucuronide (ELGlu)

ED and EL were purchased from Sigma-Aldrich (St. Louis, MO, USA). EDGlu and ELGlu were synthesized according to previous reports [29,30], with modification. Briefly, 100 µM ED aglycone or EL aglycone was incubated with 10 mg/mL mouse liver homogenates, 0.02% Triton X-100 (Nacalai Tesque, Kyoto, Japan), 1.25 mM uridine diphosphate glucuronic acid (Nacalai Tesque), 10 mM D-saccharic acid 1,4-lactone (Cayman Chemical, Ann Arbor, MI, USA), 10 mM MgCl_2_ (Nacalai Tesque), and 1 mM dithiothreitol (Wako Chemicals, Richmond, Virginia, USA) in 0.15 M Tris-HCl buffer (pH 7.4) at 37 °C for 1 h. The reaction buffer was mixed with an equal volume of methanol and two times volume of chloroform (Wako Chemicals) and vortexed to extract EDGlu or ELGlu into the aqueous phase. The aqueous phase was collected, and EDGlu and ELGlu were purified using an HPLC system (Chromaster; Hitachi High-Tech, Tokyo, Japan) coupled with an ODS column (Intertsil ODS-3, 6 µm, 4.6 × 150 mm; GL Science, Tokyo, Japan). Samples were eluted using a mobile phase composed of (A) water/acetonitrile/acetate (95:5:0.1, *v*/*v*) and (B) acetonitrile at 1.5 mL/min. The gradient program was started at 100:0 (A:B), ramped to 78:22 after 0 min, to 30:70 after 11 min, held for 9 min, ramped to 100:0 after 20 min, and held for 5 min. EDGlu and ELGlu were detected by UV absorbance at 280 nm and fractionated. The collected solution was dried, dissolved in methanol, and used for the measurement.

### 2.2. Subject and Sample Collection

Biological samples were collected from 222 Japanese volunteers. Main cohort consists of 136 Japanese subjects (males, *n* = 51, females, *n* = 85, age: 22–66 years old) recruited at a health examination site at Kenko Labo Station, a nonprofit organization (Osaka, Japan), and Nippn Corporation (Kanagawa, Japan). Validation cohort consists of 86 Japanese subjects (males, *n* = 45, females, *n* = 41, age: 25–66 years old) recruited at Shunan City (Yamaguchi, Japan). Informed consent was obtained from all participants. This study was conducted in accordance with the Declaration of Helsinki and approved by the Ethnic Committee of NIBIOHN (approved number: 154-10, 177-09, 272-01) and by the Ethics Committee of the Nippn Corporation (approved number: 20-01) and adhered to all guidelines.

Fecal samples were collected, mixed with 3 mL of guanidine thiocyanate solution (Techno Suruga Laboratory, Shizuoka, Japan), and stored at 4 °C [31,32]. Blood was collected, and prepared serum samples were stored at −80 °C until use.

### 2.3. Analysis of Enterolignans in Serum

Serum samples were mixed with an equal volume of methanol containing 2% formic acid. After vortexing and centrifugation, the supernatant was collected and mixed with an equal volume of pure water containing 1% formic acid. The sample was mixed, filtered with a GL Chromatodisc 13N (GL Science), and used for analysis. To establish an evaluation method for enterolignans by LC-MS/MS analysis, we used EDGlu, ELGlu, ED, and EL as standards for detection in human serum. Enterolignan analysis was performed using an ultra-high performance LC (UPLC) system (ACQUITY, Waters, Milford, MA, USA) coupled with a 1.7 µm, 1.0 × 150 mm ACQUITY UPLC BEH C18 column (Waters) and a Fourier transform (FT) mass spectrometer (Orbitrap ELITE; Thermo Fisher Scientific, Waltham, MA, USA). The mobile phases were (A) water/acetate (100:0.1, *v/v*) and (B) acetonitrile, and the gradient programs were as follows: starting at 95:5, ramping to 75:25 (0–10 min), to 60:40 (10–20 min), to 10:90 (20–24 min), to 95:5 (24–25 min), and holding for 5 min. The sample was eluted at 100 µL/min. Mass spectrometric analysis was performed using FT-based multiple reaction monitoring detection. Representative chromatograms are shown in Supplementary Figure S1. Data were analyzed using Xcalibur software (version 2.2, Thermo Fisher Scientific). Analytical conditions were determined using standard products and applied to the samples.

### 2.4. Gut Microbiome Analysis

DNA extraction and 16S rRNA gene amplicon sequencing were performed as previously described [31,32]. Fecal samples mixed with guanidine thiocyanate solution were disrupted by the bead beating method using 0.1 mm glass beads. DNA was extracted with Gene Prep Star PI-80X (Kurabo Industries, Osaka, Japan), and the V3-V4 region of the 16S rRNA gene was amplified using KOD-Plus-V2 (Toyobo, Osaka, Japan) with the following primers: forward, 5-TCGTCGGCAGCGTCAGATGTGTATAAGCGACAGCCTACGGGNGGCWGCAG-3, and reverse, 5-GTCTCGTGGGCTCGGAGATGTGTATAAGAGACAGGACTACHVGGGTATCTAATCC-3. Amplicons were sequenced by the paired-end method using MiSeq (Illumina, San Diego, CA, USA).

All steps from FASTQ file trimming to gut microbiome diversity analysis were automatically performed according to previously described methods [33]. The paired-end FASTQ data were trimmed and merged before the selection of operational taxonomic units (OTUs). OTU classification and diversity analyses were performed using the QIIME pipeline (version 1.9.1) [34]. The OTUs were clustered against the SILVA 128 reference database [35] at 97% similarity using the USEARCH algorithm [36]. Taxonomic classification was performed using the SILVA 128 reference database until the genus level. Taxonomy names were expressed as a specific taxonomy name based on the SILVA database phylogenetic classification standard “https://www.arb-silva.de/browser/ssu/ (accessed on 24 February 2021)”.

### 2.5. Statistical Analysis

The output of the QIIME pipeline in the BIOM table format was imported and analyzed in R (version 3.5.1) [33]. Alpha diversity indices were calculated using the estimate richness function in the phyloseq R package [37]. Principal coordinate analysis (PCoA) was performed using the *dudi.pco* function in the ade4 R package [38]. PERMANOVA was conducted using the *adonis2* function in the vegan R package [39]. We used the Wilcoxon Rank-Sum test (*wilcox.test* function in the stats R package [40]) for comparison analysis. LEfSe analysis was performed by calculating the linear discriminant analysis effect size and ranking based on this value to identify differentially abundant bacterial taxa among the groups [41]. The random forest classification model was performed using the random forest, caret, and epi R packages [42,43]. Repeated cross-validation was performed to account for the small sample size and improve the evaluation of the model. The number of folds was 10 and the number of repetitions was 10. For hyperparameters, the ntree was set to 500, and mtry was tuned using the caret R package [43]. The default values of the random forest R package were used for the other parameters. The importance of each variable was calculated using the *varImp* function in the caret R package [43]. Box plots and PCoA figures were created using the R package ggplot2 [44]. All statistical tests were two-sided with a significance level of *p* < 0.05.

## 3. Results

### 3.1. Detection of Enterolignans and Definition of Subject’s Enterolignan Metabolic Profile

**Figure 1:**
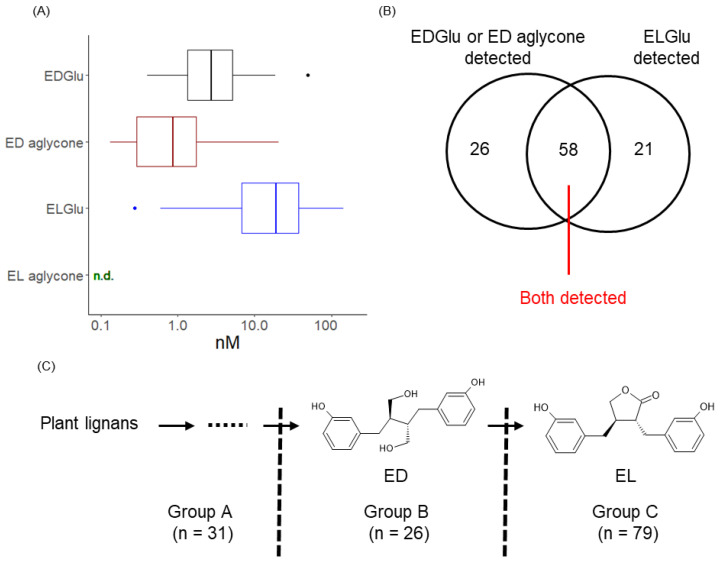
Detection of enterolignans in human serum and evaluation of individual enterolignan metabolic profile in Main cohort. (**A**) Concentration of EDGlu, ED, ELGlu, and EL in human serum. In the box plots, the boundary of the box closest and farthest to zero indicates the 25th and 75th percentile, respectively, and a line within the box marks the median. Whiskers indicate minimum and maximum values. Points above the whiskers indicate outliers. (**B**) Distribution of enterolignan metabolic profiles in Main cohort. (**C**) Discrimination of subjects in Main cohort based on individual enterolignan metabolic profile. Subjects were divided into Group A (who cannot produce either ED or EL), Group B (who can produce ED but not EL), and Group C (who can produce EL, including people who can produce ED also).

We first analyze serum enterolignans using human serum obtained from Main cohort. According to previous reports, enterolignans mainly circulate in a glucuronide-conjugated form, with a small portion of aglycone in the blood [45,46], although detection ratios of conjugates and aglycones are different because of the difference in conversion rates among molecules [47]. To establish an evaluation method for enterolignans, we prepared enterolignan glucuronides EDGlu and ELGlu as described in the Methods, and used them as standards for LC-MS/MS analysis to detect enterolignans in human serum. Consistent with previous reports [45,46], ED was detected as both EDGlu and ED aglycone, whereas EL was detected as ELGlu only (Figure 1A). Based on these results, we defined the subject’s enterolignan metabolic profile as ED producer (ED aglycone or EDGlu found) and EL producer (ELGlu found). Neither ED nor EL was detected in the 31 subjects, ED but not EL was detected in 26 subjects, EL but not ED was detected in 21 subjects, and both ED and EL were detected in 58 subjects (Figure 1B). This result indicated the individual differences in the ability to produce enterolignans. Based on these results, the subjects were divided into three groups: Group A, subjects with neither ED nor EL detected (*n* = 31); Group B, subjects with ED but not EL detected (*n* = 26); and Group C, subjects with EL detected, including subjects who also had ED detected (*n* = 79) (Figure 1C). The percentage of non-enterolignan producers (Group A) was 22.8%, of only ED producer (Group B) was 19.1%, of EL producer (Group C) was 58.1% (Figure 1C), and of enterolignan producer (Group B+C) was 77.2%.

### 3.2. Gut Microbiome Composition Is Associated with Enterolignan Metabolic Profile

**Figure 2:**
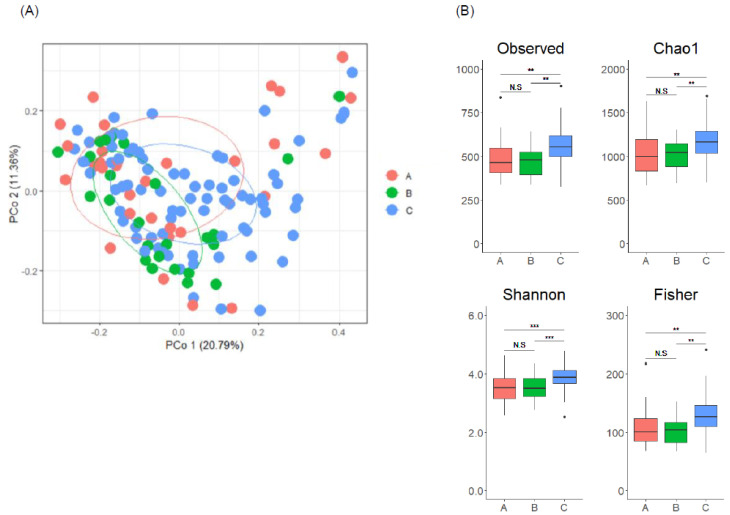
Gut microbiome characteristics differ depending on enterolignan metabolic profile. (**A**) PCoA of gut microbiome based on genera abundance between Groups A, B, and C. Statistical significance was found among the three groups by PERMANOVA (Pr = 0.0021). (**B**) Alpha diversity indices of each group. In the box plots, the boundary of the box closest and farthest to zero indicates the 25th and 75th percentile, respectively, and a black line within the box marks the median. Whiskers indicate minimum and maximum values. Points above the whiskers indicate outliers. Statistical significance was evaluated by Kruskal-Wallis test followed by Bonferroni’s multiple comparison test. N.S; not significant, ** *p* < 0.01, and *** *p* < 0.001.

We next analyzed gut microbiome using fecal samples collected from Main cohort and compared their characteristics among the three groups. PCoA analysis revealed significant differences between the groups (Figure 2A, PERMANOVA, Pr = 0.0021). We also found that alpha diversity indices were significantly higher in Group C than in the other groups (Figure 2B). These results suggest an association between enterolignan metabolic profile and gut microbiome composition.

### 3.3. Gut Microbiome Characteristics of Enterolignan Producers

**Figure 3:**
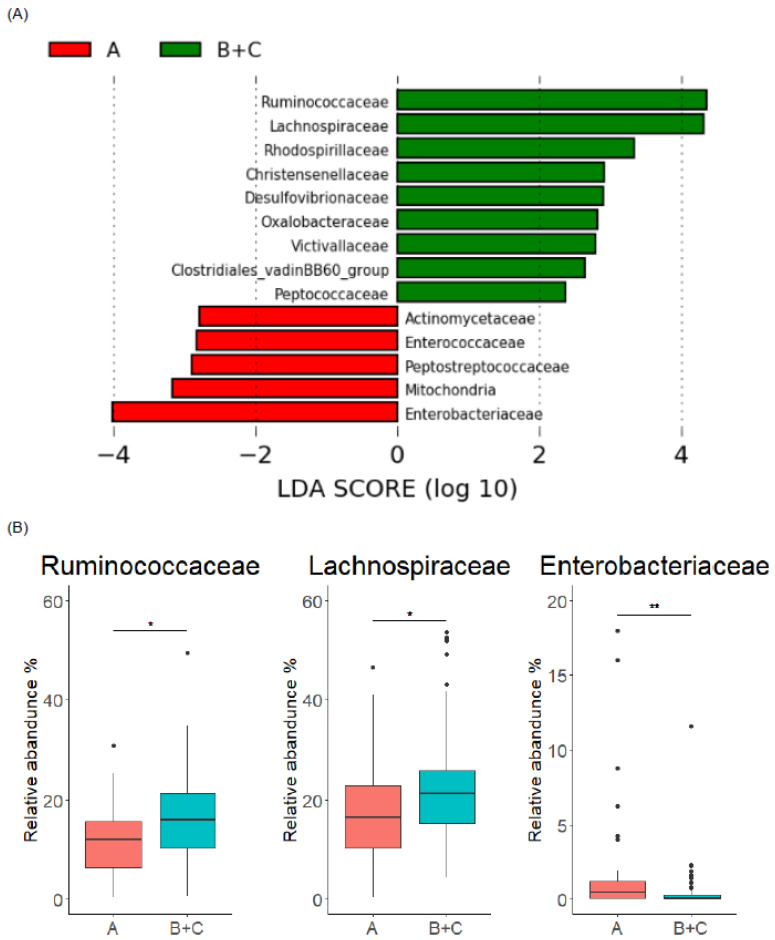
Comparison of gut microbiome composition at family level between enterolignan producers and non-producers. (**A**) LEfSe analysis at family level of enterolignan producers (Group B + C) and non-producers (Group A). (**B**) Comparison of relative abundance of Ruminococcaceae, Lachnospiraceae, and Enterobacteriaceae between enterolignan producers (Group B + C) and non-producers (Group A). In the box plots, the boundary of the box closest and farthest to zero indicates the 25th and 75th percentile, respectively, and a black line within the box marks the median. Whiskers indicate minimum and maximum values. Points above the whiskers indicate outliers. Statistical significance was evaluated by Mann-Whitney U test. * *p* < 0.05, and ** *p* < 0.01.

Next, we analyzed the relationship between individual enterolignan metabolic profile and gut microbiome composition in detail. In the gut microbiome composition between enterolignan producers (Group B and C, *n* = 105) and non-producers (Group A, *n* = 31), LEfSe analysis indicated that Ruminococcaceae and Lachnospiraceae were significantly abundant in producers at the family level, whereas Enterobacteriaceae was abundant in non-producers (Figure 3A,B). At the genus level, enterolignan producers showed increased levels of bacterial genera in Ruminococcaceae (*Subdoligranulum*, *Ruminococcus.1*, etc.) and Lachnospiraceae (*Roseburia*, *Lachnospiraceae.NK4A136.group*, etc.), while bacterial genera in Enterobacteriaceae (*Klebsiella*) were abundant in non-producers (Supplementary Figure S2A,B). These results indicate that enterolignan production could be determined by the abundance of Ruminococcaceae and Lachnospiraceae (for producer), and Enterobacteriaceae (for non-producer).

### 3.4. Gut Microbiome Characteristics of EL Producers

**Figure 4:**
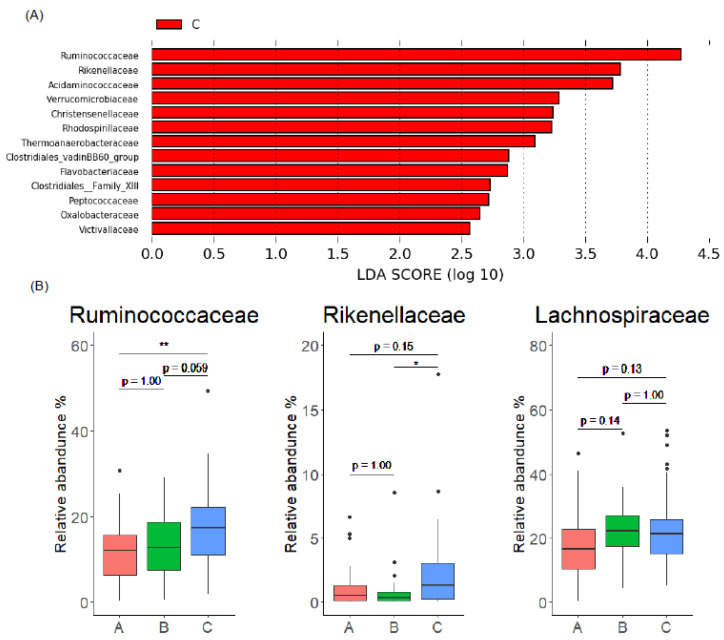
Comparison of gut microbiome composition at family level between EL producers and non-producers. (**A**) LEfSe analysis at family level comparing EL producers (Group C) and EL non-producers (Group B). (**B**) Comparison of relative abundance of Ruminococcaceae, Rikenellaceae, and Lachnospiraceae of EL producers (Group C) and other groups. In the box plots, the boundary of the box closest and farthest to zero indicates the 25th and 75th percentile, respectively, and a black line within the box marks the median. Whiskers indicate minimum and maximum values. Points above the whiskers indicate outliers. Statistical significance was evaluated by Kruskal-Wallis test followed by Bonferroni’s multiple comparison test. * *p* < 0.05, and ** *p* < 0.01.

We next attempted to identify the gut microbiome composition of EL producers. Comparison of gut microbiome between Group B and C using LEfSe analysis showed that a variety of bacteria were enriched in Group C (Figure 4A and Supplementary Figure S3A). Among them, Ruminococcaceae (*Ruminococcaceae.UCG.002*, *Ruminococcus.1*) and Rikenellaceae (*Alistipes*) were abundant in Group C (Figure 4B and Supplementary Figure S3A,B). Although a similar level of abundance was noted between Group B and C in Lachnospiraceae (Figure 4B), which was abundant in enterolignan producers (Figure 3B), the genus *Blautia* was decreased in Group C, while other bacteria in Lachnospiraceae (*Lachnospiraceae.NK4A136.group*, *Fusicatenibacter*, etc.) increased in Group C (Supplementary Figure S3A,B). A reduction of *Blautia* in EL producers has also been observed in a previous study [25], as has an increase in some bacterial genera in Lachnospiraceae in EL producers [25]. Thus, the difference in Lachnospiraceae composition, including the replacement of *Blautia* with other Lachnospiraceae genera, might be one of the gut microbiome characteristics of EL producers. Collectively, these results indicate that the gut microbiome composition of EL producers is highly diverse and rich in Ruminococcaceae and Rikenellaceae, with the replacement of *Blautia* with other Lachnospiraceae genera.

### 3.5. Gut Microbiome-Based Classification of Enterolignan Metabolic Profile

**Figure 5:**
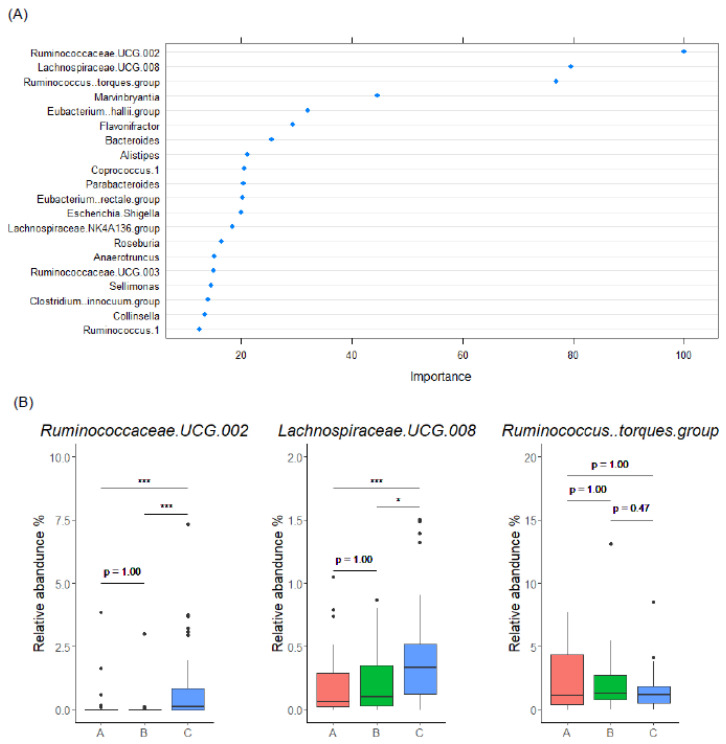
A random forest model to classify EL producers based on microbial genus data. (**A**) The top 20 explanatory variables that are important for EL producer classification (Model 1). (**B**) Relative abundance of the top 3 explanatory variable bacteria. In the box plots, the boundary of the box closest and farthest to zero indicates the 25th and 75th percentile, respectively, and a black line within the box marks the median. Whiskers indicate minimum and maximum values. Points above the whiskers indicate outliers. Statistical significance was evaluated by Kruskal-Wallis test followed by Bonferroni’s multiple comparison test. * *p* < 0.05, and *** *p* < 0.001.

Based on the association between gut microbiome composition and enterolignan metabolic profile, we created a random forest model using Main cohort to classify the subjects to EL producer and non-producer. That model successfully classified EL producers (Group C) and non-producers (Groups A and B) using the gut microbiome abundance data at genus level with an accuracy of 0.758 ± 0.054 (mean ± SD) and an area under the curve (AUC) of 0.747 ± 0.061 (mean ± SD) (Table 1, Model 1). Classification Model 1 also identified essential explanatory variables, such as *Ruminococcaceae. UCG.002*, *Lachnospiraceae. UCG.008*, and *Ruminococcus.torques. group* (Figure 5A,B). *Ruminococcaceae. UCG.002* and *Lachnospiraceae. UCG.008* were increased in Group C subjects compared with levels in Group A and B subjects (Figure 5B), which was consistent with a rank of LEfSe analysis (Supplementary Figure S3A). Furthermore, classification Model 2, which uses these three genera, showed comparable accuracy and AUC with that of Model 1, an accuracy of 0.762 ± 0.051 (mean ± SD) and an AUC of 0.760 ± 0.048 (mean ± SD), indicating the importance of these three genera (Table 1). Overall, we identified three explanatory variables that may be used to construct a random forest classification model to determine the presence or absence of EL production.

Next, in order to confirm the validity of this classification model (Model 2) to another cohort, we prepared the data obtained from other Japanese cohort (Validation cohort; Group A (*n* = 38), Group B (*n* = 17), and Group C (*n* = 31)) (Supplementary Figure S4A). Although resident area of subjects in Validation cohort was different to that of subjects in Main cohort, Model 2 can classify EL productivity with an accuracy of 0.709 and an AUC of 0.709. In addition, the increase in *Ruminococcaceae. UCG.002* and increasing trend of *Lachnospiraceae. UCG.008* in EL producer were also observed (Supplementary Figure S4B) that were consistent with Main cohort (Figure 5B). Based on this result, we confirmed the applicability of our Model 2 to the other cohort.

## 4. Discussion

Although an association between gut microbiome and enterolignan metabolic profile has been reported, it is yet to be verified in a Japanese population to the best of our knowledge. Because Japanese population generally consume plant lignans and have considerably different gut microbiome from those of people in other countries, we expected that some associations between enterolignan production and gut microbiome characteristics could be further and better elucidated by a study on Japanese population. In this study, we evaluated the relationship between enterolignan metabolic profile and gut microbiome composition in the Japanese population aged 22–66 years old. In addition, we identified explanatory variable bacteria in EL producers and constructed a gut microbiome-based machine learning model to classify the presence or absence of EL production.

We found that over half of the subjects produced enterolignans (ED and/or EL) and nearly half of the subjects produced EL, indicating individual differences in enterolignan productivity in Japanese population. In previous studies conducted in different countries, the percentage of producers in study participants ranged from 75 to 100% for ED producers and from 39 to 97% for EL producers [17,48,49,50], although not all studies described the percentages of enterolignan producers among participants, and exact protocols differed. These results indicate that differences in individual enterolignan productivity exist even among people of different residential areas, ethnicities, and dietary habits.

We next attempted to identify the association between enterolignan metabolic profile and gut microbiome characteristics in Japanese population. A previous study comparing the gut microbiome composition between Japan and other 11 countries found that Japanese gut microbiome was more abundant in *Bifidobacterium* and *Blautia* than those of other nations [27]. On the other hand, in other studies targeting Japanese people, gut microbiomes that were rich in *Bifidobacterium* or *Blautia* showed lower alpha diversity indices than microbiomes enriched in other types of gut microbiomes [51]. Because higher alpha diversity indices were characteristic of the gut microbiomes of EL producers in Japanese people, we speculated that *Bifidobacterium* or *Blautia* abundance was not related to EL productivity. Indeed, an association of *Bifidobacterium* with enterolignan metabolism was not detected in our study, and the relative abundance of *Blautia* was not higher in EL producers.

We showed that the gut microbiomes of Japanese EL producers are rich in Ruminococcaceae and Rikenellaceae. In a previous study among Japanese population, a relationship between relative abundance of Ruminococcaceae and alpha diversity indices was detected [51]. In addition, a certain percentage of Japanese population have Ruminococcaceae-abundant gut microbiomes regardless of resident area [52], and alpha diversity index is not different among individuals aged above 20 [53]. These results indicate that a certain proportion of Japanese population have EL-producing gut microbiomes across different regions and ages.

Since EL producer’s gut microbiome characteristics have been reported from some nations, we also found similarities and differences between Japanese and non-Japanese cohort. First of all, EL producers have highly diverse gut microbiome in Japanese population and this trend was also observed in United States and United Kingdom [24,25,26]. Next, we observed higher abundance of Ruminococcaceae and Rikenellaceae in EL producer in Japanese cohort and similar trend was reported in United States and Germany [25,49,54]. In addition, *Ruminococcaceae. UCG.002* genera was abundant in EL producer in two Japanese cohort and this trend was also observed in EL producer in United States [25]. These results indicate that gut microbiome diversity and high abundance of Ruminococcaceae, Rikenellaceae and *Ruminococcaceae. UCG.002* genera are robust characteristics of EL producer’s gut microbiome regardless of ethnicity. In contrast, some different characteristics were also found. For example, an association between EL production and *Lachnospiraceae. UCG.008* was found in our Japanese cohort but not reported in non-Japanese cohorts. We also elucidated *Ruminococcus.torques. group* genera as an explanatory variable for classifying EL producer, while this association was not reported in non-Japanese cohorts. Based on these results, we speculate that these two genera were unique characteristics of Japanese EL producer.

In previous studies aimed at identifying EL-producing bacteria, *Lactonifactor longoviformis* was found to be capable of converting ED to EL. However, it has been reported that the abundance of *Lactonifactor longoviformis* is relatively low in the gut, although a certain percentage of people can produce EL [15,16,50]. These results indicate the presence of other bacteria that can convert ED to EL. As an association between EL productivity and gut microbiome characteristics (e.g., diversity and abundance of Ruminococcaceae) has been elucidated in previous studies and reproduced in our results, a diverse gut microbiome is more likely to comprise microorganisms containing enzymes that catalyze conversion of ED to EL. In this study, we identified several bacteria associated with enterolignan metabolic profile, as well as three highly important explanatory variable bacterial genera for classification of EL producers. In order to clarify their involvement on EL production in detail, it is important to investigate functional characteristics of these genera. In a previous study, EL producer had higher potential to convert primary bile acid to secondary metabolites than EL non-producer in the gut [17]. In addition, fecal bacterial genes of EL producer showed high expression of bile acid-inducible gene cluster and hydroxysteroid dehydrogenases [17]. Therefore, we believe that EL-producing bacteria may have various unique pathways and some of these pathways may involve in enterolignan metabolism. Further studies are needed to evaluate functional characteristics of EL-producing bacteria in detail using several approaches such as meta-genomics, isolation of specific bacteria, and culturing them with substrate.

As a result of random forest machine learning, a classification model dividing EL producers and non-producers was constructed using either all genera or the three explanatory genera. Machine learning system has been used to evaluate associations between health efficacy of dietary nutrient and gut microbiome. For example, the efficacy of dietary barley intake on dyslipidemia could be discriminated by gut microbiome-based random forest classification [55]. Short-chain fatty acids produced from dietary fiber were elucidated as key mediators of health benefits of the Mediterranean diet through a random forest classification analysis [13]. As shown in these and our current studies, machine learning-based approach may be useful to predict the efficacy of dietary nutrients for each subject, which leads to the realization of precision nutrition. Since EL productivity of subjects can be easily classified using our classification model, it is expected to predict the availability and health efficacy of plant lignan for each subject. Although this classification model is applicable to other Japanese population with a certain accuracy, further verification is required to check whether this model is broadly applicable to other cohort with different subject characteristics such as dietary habit and ethnicity. In addition, further investigations, such as intervention studies, should be performed to determine whether it is possible to predict an individual EL productivity using gut microbiome data. After these investigations are completed, a prediction program could be developed, enabling easy analysis of individual EL productivity.

This study has several limitations. First, because this was not an intervention-based study, we could not consider some factors (e.g., individual intake of plant lignans, pharmacokinetics) that may affect the presence of enterolignans in serum and the definition of the subject’s lignan productivity; therefore, further intervention studies are warranted to establish a relationship between enterolignan productivity and gut microbiome. Second, we did not directly evaluate enterolignan productivity using fecal lysates from the subjects. Third, the number of subjects was small (Main cohort; males *n* = 51, females *n* = 85, Validation cohort; males *n* = 45, females *n* = 41), especially in Group A and B; therefore, further studies with a larger sample size are required to confirm our data. It is also important to evaluate applicability of our current classification model to non-Japanese cohort although validation to Japanese cohort was checked. In addition, because we used 16s rRNA data for analysis, we could not analyze functions of newly identified genera and its association to enterolignan production. In order to evaluate the functional characteristics of these genera in detail, further studies such as meta-genome analysis, isolation of specific bacteria and culturing bacteria with lignans are needed. Finally, it should be noted that our classification model was not validated for use in predicting individual enterolignan metabolic profile. Overall, further investigations are needed to clarify the causal relationships between gut microbiome composition and lignan metabolism through intervention-based trials and enable gut microbiome-based prediction of enterolignan metabolic profile.

In this study, we revealed the relationship between enterolignan metabolic profile and gut microbiome in the Japanese population. In addition, we elucidated some microbial features related to enterolignan metabolic profile that may be common among ethnicities. Finally, we determined that *Ruminococcaceae. UCG.002*, *Lachnospiraceae. UCG.008*, and *Ruminococcus.torques. group* are associated with EL production, and we successfully classified individual EL productivity depending on the abundance of these bacteria using random forest. This information can be used to construct a machine learning model that can predict a subject’s lignan availability without intervention. Our results provide new perspectives on the association between plant lignan availability and efficiency of their metabolism.

## Figures and Tables

**Table 1:** Results of random forest classification model for individual EL productivity.

	Accuracy	AUC
Model 1: training data set	0.741 ± 0.025	0.712 ± 0.018
Model 1: test data set	0.758 ± 0.054	0.747 ± 0.061
Model 2: training data set	0.780 ± 0.027	0.760 ± 0.027
Model 2: test data set	0.762 ± 0.051	0.760 ± 0.048

Model 1: Classification for Groups A + B, and C. Model 2: Classification by top three important genera for Groups A + B, and C. Data are shown as Mean ± SD (*n* = 10).

## Data Availability

DNA sequencing data generated in this study have been deposited in the DNA Databank of Japan (DDBJ) Sequence Read Archive under the accession number DRA014928, DRA014929, and DRA015021.

## Supplementary Materials

The following supporting information can be downloaded at: https://www.mdpi.com/article/10.3390/microorganisms10112169/s1, Supplementary Figure S1. Representative LC-MS/MS multiple reaction monitoring chromatograms of EDGlu, ED, ELGlu, and EL standard. Supplementary Figure S2. Comparison of gut microbiome composition between enterolignan producers (Groups B + C) and enterolignan non-producers (Group A) at genus level. Supplementary Figure S3. Comparison of gut microbiome composition between EL producers and EL non-producers at genus level. Supplementary Figure S4. Validation of the constructed random forest classification model for EL producers using Validation cohort.
