# Supplementary material for: Identification of Human Gut Microbiome Associated with Enterolignan Production
Source: Microorganisms. 2022 Oct 31;10(11):2169. doi: 10.3390/microorganisms10112169 (PMC9697123; doi:10.3390/microorganisms10112169)
Supplement: Supplementary file 1 [file microorganisms-10-02169-s001.zip › microorganisms-1965140-supplementary.pdf]

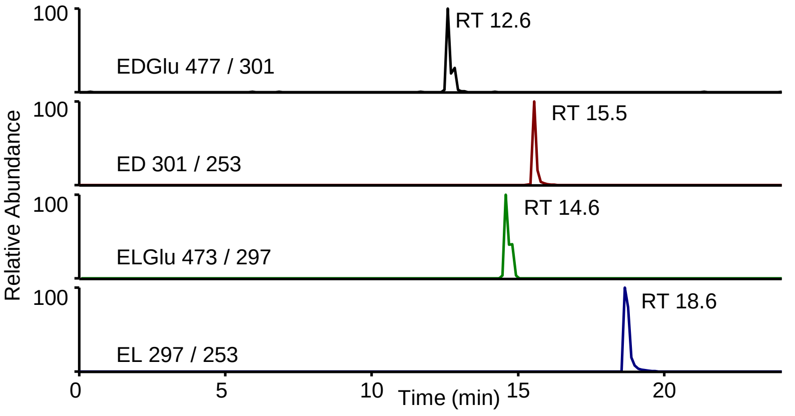

Supplementary Figure S1. Representative LC-MS/MS multiple reaction monitoring chromatograms of EDGlu, ED, ELGlu, and EL standard.

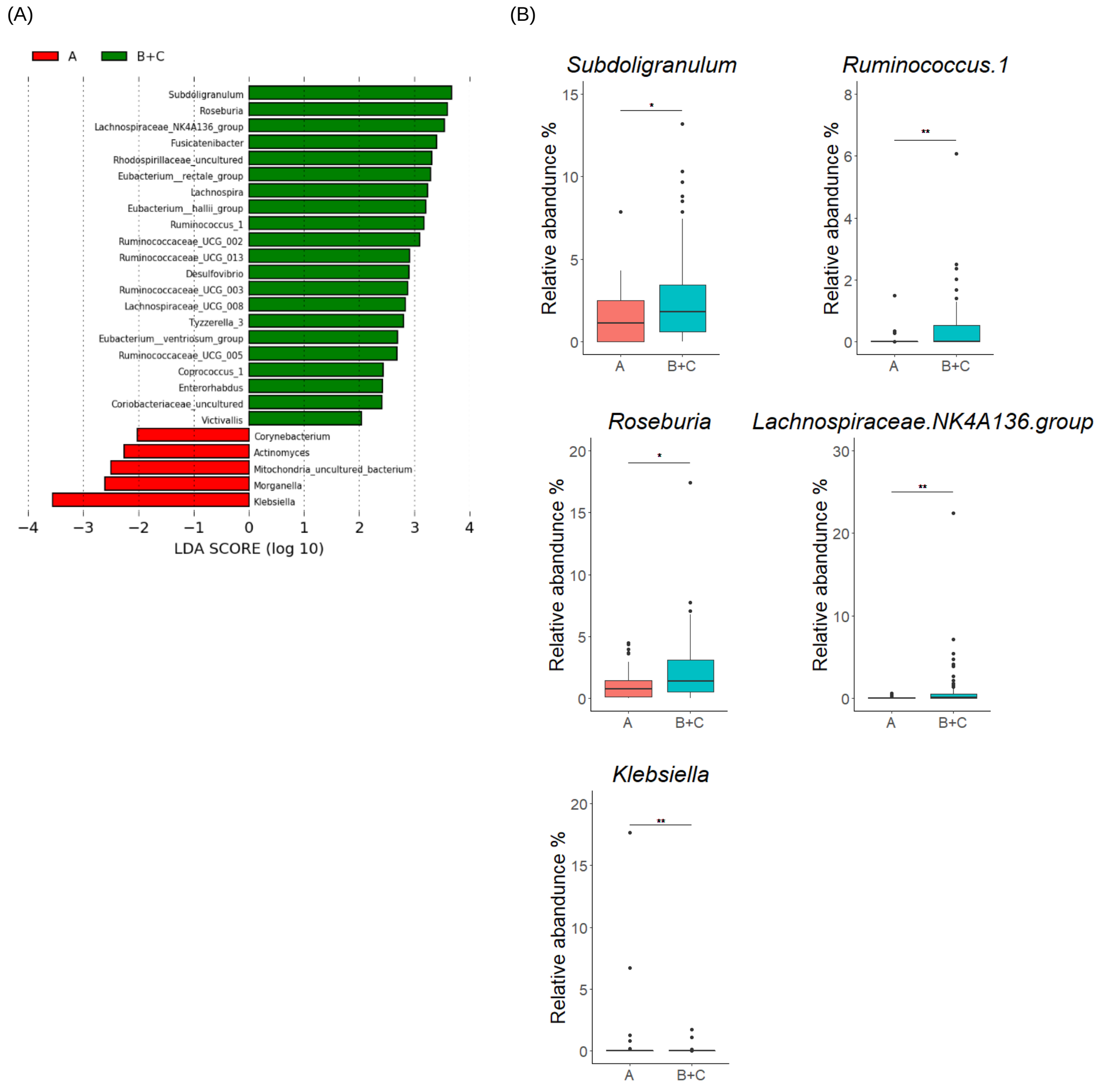

Supplementary Figure S2. Comparison of gut microbiome composition between enterolignan producers (Groups B + C) and enterolignan non-producers (Group A) at genus level.

(A) LEfSe analysis at the genus level comparing enterolignan producers (Groups B + C) and non-producers (Group A). (B) Comparison of the relative abundance of bacteria genera in Ruminococcaceae (*Subdoligranulum*, and *Ruminococcus.1*), Lachnospiraceae (*Roseburia*, and *Lachnospiraceae.NK4A136.group*), or Enterobacteriaceae (*Klebsiella*) between enterolignan producers (Groups B + C) and non-producers (Group A). In the box plots, the boundary of the box closest and farthest to zero indicates the 25th and 75th percentile, respectively, and a black line within the box marks the median. Whiskers indicate minimum and maximum values. Points above the whiskers indicate outliers. Statistical significance was evaluated by Mann–Whitney U test. * $p < 0.05$, and ** $p < 0.01$.

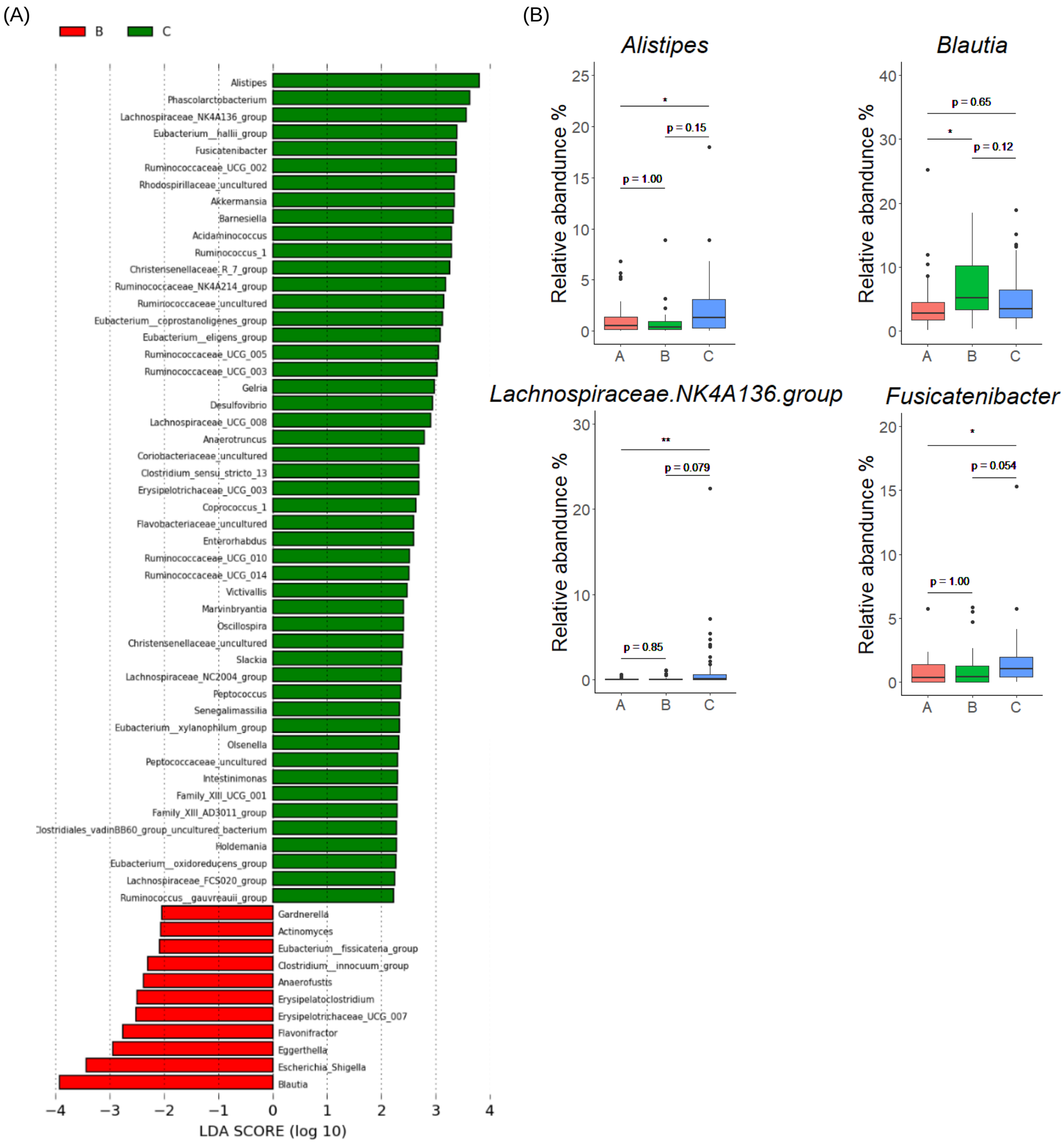

Supplementary Figure S3. Comparison of gut microbiome composition between EL producers and EL non-producers at genus level.

(A) LEfSe analysis at the genus level comparing EL producers (Group C) and EL non-producers (Group B). (B) Comparison of the relative abundance of bacteria genera in Rikenellaceae *(Alistipes)*, and Lachnospiraceae (*Blautia*, *Lachnospiraceae.NK4A136.group*, and *Fusicatenibacter*) of EL producers (Group C) and other groups. In the box plots, the boundary of the box closest and farthest to zero indicates the 25th and 75th percentile, respectively, and a black line within the box marks the median. Whiskers indicate minimum and maximum values. Points above the whiskers indicate outliers. Statistical significance was evaluated by Kruskal-Wallis test followed by Bonferroni's multiple comparison test. * $p < 0.05$, and ** $p < 0.01$.

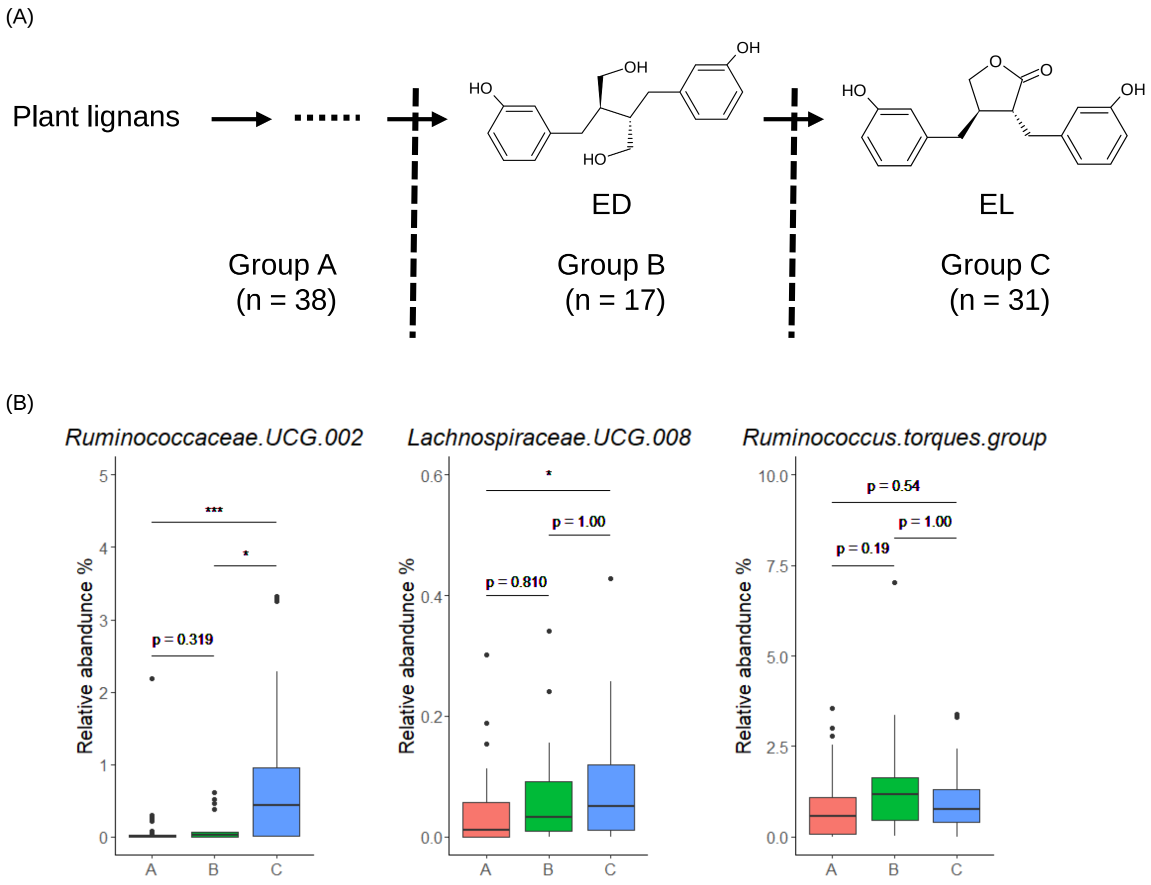

Supplementary Figure S4. Validation of the constructed random forest classification model for EL producers using Validation cohort.
(A) Discrimination of subjects in Validation cohort based on individual enterolignan metabolism. Subjects were divided into Group A (who cannot produce either ED or EL), Group B (who can produce ED but not EL), and Group C (who can produce EL, including people who can produce ED also). (B) Relative abundance of the explanatory variable bacteria in Validation cohort. In the box plots, the boundary of the box closest and farthest to zero indicates the 25th and 75th percentile, respectively, and a black line within the box marks the median. Whiskers indicate minimum and maximum values. Points above the whiskers indicate outliers. Statistical significance was evaluated by Kruskal-Wallis test followed by Bonferroni's multiple comparison test. * $p < 0.05$, and *** $p < 0.001$.
